# Chinese college students’ PGD symptoms and their relationship to cognitive variables: a latent profile analysis

**DOI:** 10.3389/fpsyg.2024.1242425

**Published:** 2024-04-23

**Authors:** Weicui Tian, Yang Cui, Meiling Liao, Fajie Huang

**Affiliations:** ^1^School of Public Health and Health Management, Fujian Health College, Fuzhou, China; ^2^School of Health, Fujian Medical University, Fuzhou, China

**Keywords:** PGD, latent profile analysis, irrational beliefs, basic world assumptions, CBT

## Abstract

Bereavement is a commonly experienced grief event; however, bereavement can also trigger a number of psychological consequences, such as prolonged grief disorder (PGD). At present, the differences in prolonged grief disorder symptoms (PGD symptoms) among various individuals and how those symptoms relate to cognitive variables are unclear. In the present study, 817 Chinese college students with bereavement experience were selected as participants. Based on the evaluation results of their irrational beliefs, bereavement-related irrational beliefs, basic world assumptions, and PGD symptoms, an individual-centered latent profile analysis was used to divide a group with PGD symptoms into several subgroups and comprehensively examine the relationships between these subgroups and cognitive variables. (1) The severity of PGD symptoms among Chinese college students can be categorized into three subgroups: mild, moderate, and severe. (2) Cognitive variables such as irrational beliefs and basic world assumptions were all found to correlate with the severity of PGD symptoms; bereavement-related irrational beliefs was the variable with the largest correlation. However, for the first time, this study found that different dimensions of basic world assumptions had different directions of correlation, based on the severity of the PGD symptoms. Justice, control, randomness, and self-control had significantly positive correlations. Conversely, benevolence of the world, benevolence of people, and worthiness of the self had significantly negative correlations. These results have important reference value for cognitive behavioral therapy (CBT) treatment and interventions for PGD issues in Chinese college students.

## Introduction

Bereavement is one of the most challenging life stress time events that individuals may experience throughout the course of life development. College students are in a transitional stage from adolescence to adulthood, a period of rapid psychological and physiological maturity. Bereavement, a common major crisis event that occurs during this period, inevitably has a significant impact on future educational, life, and occupational outcomes (Mannarino and Cohen, 2014; Andriessen et al., 2019). Therefore, the grief-based psychological problems experienced by college students after experiencing bereavement have attracted much attention from Chinese mental health workers (Li et al., 2017; Boelen et al., 2021; Huang et al., 2023).

Generally speaking, after bereavement, people experience a high-intensity grief reaction in a short period of time, as demonstrated through crying, a sense of heartbreak, and so on (Kristensen et al., 2012; Szuhany et al., 2021). Later in life, people manage to adapt to bereavement without suffering as severe and lengthy grief symptoms and gradually begin to cope with life again; however, a considerable number of people have difficulties recovering from the pain of prolonged grief symptoms, and even develop psychiatric complications such as depressive- and anxiety-related symptoms. More seriously, a small number of people develop prolonged grief disorder (PGD) (Stroebe et al., 2007; Prigerson et al., 2009; Maercker et al., 2013). At present, PGD has been formally included in the International Classification of Diseases, 11th edition (ICD-11: World Health Organization [WHO], 2018) and Diagnostic and Statistical Manual of Mental Disorders 5, Text Revision (DSM-5-TR: American Psychiatric Association [APA], 2022). PGD manifests as a series of intense grief reactions that persist in a person at least 12 months after bereavement (for children and adolescents, at least six months), with persistent thoughts of and longing for the deceased and a dwelling on thoughts and memories of the deceased acting as core symptoms. These are accompanied by intense emotional distress, severely impairing the bereaved person’s day-to-day functioning. This grief response is not consistent with the social and cultural environment in which they live (Wortman and Boerner, 2011; Silverman and Rubin, 2015; Duffy and Wild, 2017; Szuhany et al., 2021).

What is the psychological mechanism fueling PGD symptoms after loss? At present, the cognitive-behavioral conceptualization model (CBCM) proposed by Boelen et al. (2006) is one such systematic psychological theory. This model holds that when an individual faces loss, the original negative cognition directly leads to a pathological grief reaction, coupled with a negative avoidance strategy. The interaction of these factors makes the grief reaction both lasting and intense, eventually evolving into PGD. In other words, the evolution of PGD symptoms in individuals is the result of the interaction of loss experience, negative cognition, anxiety, and depression, among which negative cognitive variables play the key role in developing prolonged grief symptoms (Boelen et al., 2006; Ehlers, 2006). Previous studies have shown that the impact of bereavement on individual cognitive systems mainly includes two aspects: irrational beliefs and basic world assumptions (Janoff-Bulman, 1989; Boelen et al., 2003; Rubin et al., 2016). First of all, after experiencing bereavement, individuals increase their tendency to engage in general irrational thinking, and subsequently, irrational beliefs regarding bereavement and the self are dominant, including low frustration tolerance and discomfort anxiety, making individuals more prone to PGD symptoms (Ellis, 2001; Ehlers, 2006; Nagy and Szamosközi, 2014). Secondly, after bereavement, the individual’s basic world assumptions about the self, others, and the external world are damaged. World assumptions mean the individual’s set of basic cognitive schemata, which is the core content of the individual belief system; this includes eight aspects (e.g., benevolence of the world, benevolence of people, randomness, etc.). Basic assumptions of the world are developed by the individual over many years and feature the illusion of invulnerability that is necessary for the individual to perform the activities of daily life (Janoff-Bulman, 1989). After experiencing bereavement, these stable basic world assumptions are broken, resulting in PGD symptoms (Janoff-Bulman, 1992). However, to our knowledge, there has been no quantitative study that systematically explores the relationship between the two cognitive variables of irrational beliefs (defined as individuals’ unrealistic demands that lack an objective basis, including absolute demandingness, awfulizing, low frustration tolerance, and global evaluation) and basic world assumptions (a set of basic cognitive schema that individuals have about the self and world, the content of which can be categorized into three main types: perceived benevolence of the world, meaningfulness of the world, and worthiness of the self) and their relation to PGD symptoms.

In addition, there is as yet no conclusive answer to the question of what specific cognitive variables are significantly associated with PGD symptoms (Boelen et al., 2003; Zhou et al., 2018). This uncertainty is most obvious in the cognitive variable of basic world assumptions (Schwartzberg and Janoff-Bulman, 1991; Boelen et al., 2004; Currier et al., 2009; Schuler and Boals, 2016). For example, Boelen et al. (2004) found that the PGD symptoms of Dutch college students had a significant positive correlation with irrational beliefs related to loss and a significant negative relationship with the control and luck dimensions of world assumptions. Currier et al. (2009) found that PGD symptoms in American college students had a significant negative correlation with basic world assumptions, but only the meaning and self-worth dimensions of world assumptions had significant negative correlations. As the research designs of these studies were similar, the inconsistent results between them may be due to two factors. The first is the heterogeneity of PGD symptoms of the bereaved individuals selected for each study. A bereaved population with PGD symptoms can be divided into different subgroups. The nature of this cognitive variable and severity of PGD symptoms will vary among subgroups; however, few previous studies have examined this issue. Moreover, college students reside in schools for long periods of time, and most experience a relatively limited distribution, time, and cause of loss (Ehlers, 2006; Kristensen et al., 2012; Smid et al., 2019). Thus, it would be worthwhile to explore whether PGD symptoms in college students have specific manifestations. Previous studies have mainly used the variable-centered analysis perspective. Although this perspective can reflect the overall situation of individual PGD symptoms and their relationship to other variables, it is difficult to reflect the characteristics and differences of PGD symptoms among different subgroups. Therefore, individual-centered latent profile analysis (LPA) techniques are needed (Lanza et al., 2007). By identifying the variations shared by individuals with bereavement experience and different PGD symptoms, in the present research, a heterogeneous group was divided into several homogeneous subgroups. The differences in PGD symptom characteristics among the different subgroups were then examined to solve the problem of heterogeneity in PGD symptoms.

Sociocultural factors may also affect the relationship between cognitive variables and PGD distress symptoms because variables such as individuals’ irrational beliefs and basic world assumptions are formed in a specific cultural context and obviously influenced by the norms of the individual’s cultural identity (Janoff-Bulman, 1992; Li et al., 2018). College students’ basic world assumptions about the self and world are progressively developed and refined over the course of their life experience accumulation and personal growth (Janoff-Bulman, 1989; Schuler and Boals, 2016). Importantly, the pattern of transition of PGD symptoms may also vary across cultures. For example, according to a previous study (Boelen et al., 2003; Bonanno et al., 2005), bereavement survivors in China have different patterns of grief symptoms compared with such survivors in the United States. Specifically, in the early stages of grief, Chinese bereavement survivors have stronger grief reactions and worse mental health than do such individuals in the United States. However, 18 months later, bereaved Chinese people were found to report less grief, a lower level of pain, and better mental health than did Americans. In addition, funeral culture, understanding of death, and other cultural issues are also predictors of PGD symptoms (Currier et al., 2009; Zukerman and Korn, 2014; Vermunt, 2017). Since previous studies on the psychological mechanism prompting PGD symptoms have mainly focused on bereaved people in Western countries, this study was carried out in an Eastern country (China), which has a unique funeral culture (e.g., wearing mourning clothes, sweeping graves during the Qingming Festival, etc.) and concepts related to death (e.g., rebirth, death, death taboos, etc.). Therefore, this study will improve our understanding of the relationship between PGD symptoms and cognitive variables as they operate in the Chinese culture.

In sum, this study selected Chinese college students with bereavement experience as the participants, and measured the cognitive variables (i.e., irrational beliefs and basic world assumptions) and PGD symptoms according to CBCM theory. In addition, considering the event-specific nature of individual irrational beliefs (Hyland et al., 2013), this study examined both general and bereavement-related irrational beliefs in order to more fully assess the cognitive variable of irrational beliefs in college students who have experienced bereavement. On this basis, this study used an individual-centered research method to analyze the latent profiles of PGD symptoms and determine the different latent subgroups of those symptoms in Chinese college students (Aim 1). Additionally this study used multinomial logistic regression analysis to comprehensively investigate the correlation of cognitive variables with the latent subgroups of PGD symptoms (Aim 2). The purpose of this study was to promote a more comprehensive understanding of the relationship between PGD symptoms and cognitive variables in Chinese college students with PGD.

## Materials and methods

### Participants

First, 13,965 college students from Fujian Health Vocational and Technical College, Fujian University of Traditional Chinese Medicine, Fujian Agricultural and Forestry University, Fujian Engineering College, Sunshine College, Fujian Jiangxia College, Fujian Preschool Teachers College, Zhangzhou Health Vocational College, Quanzhou Medical College, and Quanzhou Preschool Teachers College were selected as participants. A preliminary questionnaire was distributed on a class basis, and all participants were tested with the four research instruments used in this study. All tested individuals voluntarily participated and provided written informed consent beforehand. Next, on the basis of the above preliminary test and according to the previous screening criteria (Boelen et al., 2004), this study explored the relationship between PGD symptoms and cognitive variables for college students after they experienced bereavement. That is, these individuals had experienced the death of a parent, sibling, relative, or good friend in the past 0.6 to 7.6 years and have not experienced other obvious traumatic event that would be a time of obvious trauma such as rape, robbery, serious traffic accident, earthquake disaster, typhoon disaster, mudslide disaster, landslide, fire, or explosion, among others. A total of 817 college students experiencing bereavement in the past 0.6 to 7.6 years were selected as the final participant set. There were 144 boys and 673 girls in the study, totaling 817 participants; the average age was 18.79 ± 1.51 years (range = 18 to 28 years), and the time of bereavement was 2.99 ± 3.76 years from the test (range = 0.6 to 7.6 years). Additional information is shown in Table 1.

**TABLE 1:** Information about the loss of 817 participants.

Cause of death	*n* (%)	Relationship to the deceased	*n* (%)
Natural aging	261 (31.95)	Father or mother	65 (7.95)
Natural disasters (earthquakes, floods, etc.)	163 (19.95)	Relatives (grandpa, grandma, uncle, aunt, etc.)	413 (50.55)
Traffic accident	97 (11.87)	Best friend	286 (35.01)
Disease	236 (28.89)	Unwilling to reveal the subject of the death	53 (6.49)
Others (suicide, homicide, etc.)	60 (7.34)		

### Measures

All participants in this study completed four measures, including the irrational beliefs scale (IBS), bereavement-related irrational beliefs scale (BRIB), world assumptions scale (WAS), and Inventory of traumatic grief (TGI). Before each test, participants were informed that the purpose was to understand college students’ attitudes toward bereavement and death. Written informed consent was obtained and all participants participated voluntarily. In addition, the order of presentation of the four scales differed by class, so as to avoid the influence of the order of the tests and participants’ response formulas on the results.

### Irrational beliefs scale

The IBS was developed by Yang et al. (2007). It has a total of 22 items and three dimensions: low frustration tolerance (e.g., I can’t control my emotions when something is not going as expected), perfectionism (e.g., Everything I decide to do, I have to do perfectly), and global evaluation (e.g., Asking for help is a sign of weakness). The scale is mainly used to measure the general irrational beliefs of people with emotional disorders. It employs a 5-point Likert scale ranging from “strongly disagree” (0 points) to “strongly agree” (4 points). The higher the score, the higher is the degree of irrational beliefs. Since the present study mainly considered the relationship between the general level of irrational beliefs and PGD symptoms, the total IBS score was used as a variable. The results showed that the reliability and validity indicators of the IBS were good. The alpha coefficient of the total scale was 0.93 and the three sub-dimensions were between 0.87 and 0.93; confirmatory factor analysis showed that the fit indicators of the three-factor structure were good, χ2 = 648.31, df = 152, TLI = 0.91, CFI = 0.94, AIC = 44,751.56, BIC = 45,330.35, SRMR = 0.04, RMSEA = 0.063.

### Bereavement-related irrational beliefs scale

The BRIB was developed by Boelen et al. (2004). It is a single-dimensional scale with eight items, for example: “I would be a worthless person if I took too long to process this loss.” The scale was specifically designed to assess individuals’ irrational beliefs related to bereavement events. It uses a 5-point Likert scale ranging from “strongly disagree” (0 points) to “strongly agree” (4 points), with higher scores indicating higher levels of irrational beliefs related to bereavement. In the present study, the scale was translated first into Chinese, and then translated back by a researcher with an English major background. The Chinese version of the BRIB is the same as the original version in terms of the number of items and response options. The reliability and validity of the BRIB are good, alpha coefficient of the scale is 0.85, and results of confirmatory factor analysis show that the single factor structure fits well, χ2 = 86.19, DF = 17, TLI = 0.97, CFI = 0.98, AIC = 14,796.47, BIC = 119,23.53, SRMR = 0.028, RMSEA = 0.071. A significant positive correlation was found between the BRIB and IBS scores for the 817 college students with bereavement experience: *r* = 0.57, *p* < 0.001.

### World assumptions scale

The WAS was developed by Janoff-Bulman (1989). It has 32 items and is divided into three basic world assumptions and eight dimensions, including benevolence of the world assumptions (e.g., There is more good than evil in the world), benevolence of people (e.g., People are naturally unfriendly and unkind), meaningfulness of the world (e.g., Misfortune is least likely to strike worthy, decent people), control (e.g., People’s misfortunes result from mistakes they have made), randomness (e.g., The course of our lives is largely determined by chance), worthiness of self (e.g., I have a low opinion of myself), self-controllability (e.g., I usually behave so as to bring about the greatest good for me), and luck (e.g., I am basically a lucky person). The scale is mainly used to measure the individual’s basic cognition of the world and basic world assumptions. It uses a 5-point Likert score ranging from “strongly disagree” to “strongly agree.” Items 2, 8, 12, 18, and 31 are reverse-scored. The raw scores of the five reverse-scored items must be converted forward. Then, the scores of the items belonging to each dimension are added together as that dimension’s score. The scores of each dimension are then added together to form the total score for the WAS. The higher the WAS score, the higher is the level of world assumptions in the respondent’s belief system (e.g., for benevolence of people, a higher score indicates that a person’s social cognition makes them more inclined to assume that humans living in the world are friendly and kind). Huang and Zhang (2014) revised the Chinese version of the WAS. The Chinese version is consistent with the original scale in terms of the number of items, structure, and response options, and has good reliability and validity indicators. In the present study, for the α coefficient index, the total scale was 0.87 and the eight sub-dimensions were between 0.70 and 0.87. The results of a confirmatory factor analysis showed that the fit index of the second-order eight-factor structure was good, χ2 = 234.57, DF = 74, TLI = 0.96, CFI = 0.97, AIC = 11,851.01, BIC = 12,030.28, SRMR = 0.024, RMSEA = 0.074.

### Inventory of traumatic grief

The TGI was developed by Prigerson et al. (1995). It is a single-dimensional structure containing 19 items (e.g., “I cannot accept the death of the person who died,” “I feel that life is empty without the person who died,” etc.). It is mainly used to measure PGD symptoms such as the degree of separation pain (i.e., pain due to separation) and grief after loss. The scale uses a 5-point Likert scale ranging from “strongly disagree” (0 points) to “strongly agree” (4 points). The total score of the scale is calculated by adding the scores for the 19 items. The higher the score, the more serious are the PGD symptoms. Zhuang and Huang (2014) revised the Chinese version of the TGI for college students with bereavement experience. The revised TGI is consistent with the original scale in terms of the number of items and response options. Factor analysis results supported a single latent structure and had good reliability and validity indicators. In the present study, the alpha coefficient of the Chinese version of the TGI was 0.96. Confirmatory factor analysis of the single latent structure fit the indicators well, χ2 = 706.57, df = 124, TLI = 0.95, CFI = 0.93, AIC = 33,545.14, BIC = 33,945.12, SRMR = 0.05, RMSEA = 0.076. The TGI scores for college students with bereavement experience were significantly and positively correlated with irrational beliefs and bereavement-related irrational beliefs, *r*_1_ = 0.40, *p*_1_ < 0.001, *r*_2_ = 0.62, *p*_2_ < 0.001. Therefore, the measurement attributes of the Chinese version of the TGI in this study met the psychometrics requirements.

### Data analysis

Harman’s single-factor test was used to evaluate the common method bias of the research data. The results of an exploratory factor analysis showed that the variance explained by the first factor without a rotation analysis was 24.48%, which was less than the 40% critical value. This indicated that common method bias did not have a significant impact on the results of this study.

For Aim 1, Mplus8.0 software was used to analyze the latent profile of the data (Lanza et al., 2007) and explore the latent subgroups of Chinese college students’ PGD symptoms. Starting with a one-profile model, the number of profiles (or subgroups) was gradually increased and the fit indexes calculated. The measures included the likelihood ratio, Chi-squared test, log likelihood (LL), information evaluation criteria, Akaike information criterion (AIC), Bayesian information criterion (BIC) and adjusted Bayesian information criterion (aBIC). The smaller the value, the better was the model fit effect. The larger the entropy index, the higher was the classification accuracy. If entropy > 0.8, the classification accuracy of the model was more than 90%. The Lo-Mendell-Rubin (LMR) and bootstrapped likelihood ratio test (BLRT) indicated that the K-class model was significantly better than the K − 1 model if the test result was *p* < 0.05. To ensure the simplicity and interpretability of the model, the fit indicators were integrated to determine the best latent profile for fitting the model (McClintock et al., 2016).

For Aim 2, based on the LPA, the setting “AUXILIARY = X (R3STEP)” in Mplus8.0 and a robust three-step method were used to conduct a multinomial logistic regression analysis, allowing for an examination of the correlation of cognitive variables with the latent profiles of Chinese college students’ PGD symptoms (Vermunt, 2017). In the present study, a multicollinearity test was performed on the 11 predictor variables entered into the regression equation, using the three latent subgroups of PGD symptoms as the outcome variables. The results showed that the tolerance indicator for all predictors ranged from 0.39 to 0.98, and the variance inflate factor (VIF) indicator ranged from 1.02 to 3.15. This showed that there was no significant multi-collinearity for the 11 predictors.

## Results

### Latent profile analysis of Chinese college students’ PGD symptoms

In order to explore the latent subgroups of PGD symptoms experienced by Chinese college students, the scores of the 19 items measured via the TGI scale were employed as indicators for a latent profile analysis, and the latent profile models for one to six profiles successively established. The fit indicators of the models are shown in Table 2. As shown therein, the entropy values of all models were greater than 0.9, and the entropy values after Class 2 showed little change. The fit indexes of LL, AIC, BIC, aBIC, and other models showed a decreasing trend with an increase in the number of classes. Both the LMR and BLRT indicators reached significant levels, demonstrating that increasing the number of profiles may improve the model. However, after the BIC index of each profile of model was drawn into a steep slope chart, the decline in the BIC index after the third Class of model became gentle. This meant that the third profile of model was the inflection point of the change-of-fit index. In addition, compared with the fit indices for Models 3 and 4, if the individuals’ responses were divided into four latent subgroups, the probability of the smallest class was 5%, which was not conducive to classification accuracy. At the same time, responses were divided into three latent subgroups. Therefore, considering the simplicity and interpretability of the latent profile model, Model 3 was determined as the best latent profile fit model in this study. The attribution probabilities of the three latent profiles were between 96.90 and 98.90%, meaning that the classification results of the three latent profile models were reliable. See Table 3 for details.

**TABLE 2:** Indices of fit for the latent profile model of Chinese college students’ PGD symptoms (*n* = 817).

Class	LL	AIC	BIC	aBIC	Entropy	LMR (*p*-value)	BLRT (*p*-value)	Class probability
1	−22,975.19	46,026.39	46,205.21	46,084.54				
2	−19,870.21	39,856.41	40,129.34	39,945.15	0.943	0.0011	<0.001	0.66, 0.34
3	−18,669.92	37,495.84	37,862.88	37,615.18	0.944	0.0014	<0.001	0.31, 0.48, 0.21
4	−18,030.07	36,256.14	36,717.29	36,406.09	0.952	0.0017	<0.001	0.43, 0.25, 0.27, 0.05
5	−17,695.33	35,626.66	36,181.92	35,807.20	0.947	0.0314	<0.001	0.25, 0.42, 0.16, 0.06, 0.11
6	−17,369.00	35,014.00	35,663.38	35,225.14	0.935	0.3076	<0.001	0.23, 0.19, 0.01, 0.29, 0.13, 0.05

**TABLE 3:** Average attribution probabilities for three latent profiles of Chinese college students’ PGD symptoms.

	Class 1 (%)	Class 2 (%)	Class 3 (%)
Class 1	97.20	2.80	0
Class 2	2.00	96.90	1.10
Class 3	0	1.10	98.90

### Naming the three latent subgroups of Chinese college students’ PGD symptoms

**FIGURE 1:**
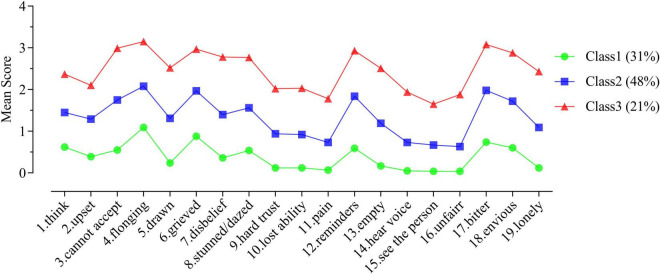
Mean scores for the 19 items representing the three latent subgroups of Chinese college students’ PGD symptoms.

Based on the results of the latent profile analysis, the mean scores of the three latent subgroups of PGD symptoms for the 19 items of the TGI scale were plotted linearly, as shown in Figure 1. The three latent subgroups of PGD symptoms did not have intersections among the items, and the morphologic trends of the different subgroups were consistent. In addition, with the three subgroups as the independent variable and total score of the TGI as the dependent variable, the results of a one-way ANOVA showed a significant main effect of the subgroups, *F*(2, 816) = 1,898.62, *p* < 0.001, ω_p_^2^ = 0.82. Multiple *post hoc* comparisons showed that the total score of Class 3 (46.79 ± 9.56) was significantly higher than that of Class 2 (25.24 ± 5.66), and the total score of Class 2 was significantly higher than that of Class 1 (7.32 ± 4.89).

Higher total TGI scores indicated more severe PGD symptoms, and the three latent subgroups were named accordingly in this study. Specifically, in Class 1, the total TGI score was the lowest among the three classes, and in addition, except for Item 4 (longing), which scored higher than 1 (rarely), the scores of all the other items were lower than 1. This suggested that Class 1 had the mildest PGD symptoms, and thus Class 1 was named the mild PGD symptom group; it accounted for 31% of the total. For Class 2, the total TGI scores were higher than for Class 1 but lower than for Class 3. Most items were between 1 (rarely) and 2 (sometimes), such as “reminders” and “bitter,” indicating that the PGD symptoms in Class 2 were at an intermediate level. Therefore, Class 2 was named the group with moderate PGD symptoms; it accounted for 48% of the total group. For Class 3, the total TGI score was the highest among the three classes. At the same time, most of the items were higher than 2 (sometimes), and some even higher than 3 (often). This indicated that the PGD symptoms in Class 3 were at a severe level. Therefore, Class 3 was named the severe PGD symptom group; this group accounted for 21% of the whole.

### Relationship between cognitive variables and latent subgroups of PGD symptoms among Chinese college students

In order to comprehensively explore the correlation of relevant cognitive variables with latent subgroups of PGD symptoms among Chinese college students, three of the subgroups were used as outcome variables, and gender, bereavement-related irrational beliefs, irrational beliefs, and underlying world assumptions were included in the 11 factors as predictor variables. A robust three-step method was used for the multinomial logistic regression analysis. ODD-ratio (OR) coefficients were calculated to indicate the magnitude of the probability that college students characterized by a predictor variable might belong to a particular latent subgroup of PGD symptoms (outcome variables), as compared to the reference group (Johnson and Lebreton, 2016). The specific results are shown in Table 4.

**TABLE 4:** Results of the multinomial logistic regression analysis of the influence of cognitive variables on the latent subgroups of Chinese college students’ PGD symptom (*n* = 817).

No.	Predictor variable	Class 2 vs. Class 1				Class 3 vs. Class 1				Class 3 vs. Class 2			
		Coefficient	SE	OR	*p*	Coefficient	SE	OR	*p*	Coefficient	SE	OR	*p*
1	Gender	-0.02	0.23	0.98	0.93	0.04	0.27	1.04	0.90	0.05	0.24	1.01	0.83
2	IBS	0.04	0.01	1.04	<0.001	0.09	0.01	1.09	<0.001	0.05	0.08	1.05	<0.001
3	BRIB	0.46	0.04	1.58	<0.001	0.23	0.03	1.26	<0.001	0.23	0.03	1.25	<0.001
4	Benevolence of the world	-0.12	0.04	0.88	0.001	0.001	0.03	1.00	0.97	-0.12	0.02	1.12	<0.001
5	Benevolence of people	-0.07	0.04	0.94	0.07	-0.28	0.04	0.76	<0.001	-0.22	0.03	0.81	<0.001
6	Justice	0.12	0.03	1.12	0.001	0.13	0.04	1.14	0.002	0.02	0.04	1.01	0.74
7	Control	0.09	0.04	1.09	0.009	0.14	0.04	1.15	<0.001	0.05	0.04	1.05	0.17
8	Randomness	0.21	0.04	1.24	<0.001	0.07	0.03	1.07	0.04	0.15	0.04	0.86	<0.001
9	Self-worth	-0.20	0.04	0.82	<0.001	-0.42	0.05	0.66	<0.001	-0.20	0.04	0.80	<0.001
10	Self-controllability	0.08	0.03	1.08	0.02	0.11	0.04	1.12	0.007	0.03	0.03	1.03	0.09
11	Luck	-0.02	0.03	0.98	0.45	-0.06	0.04	0.95	0.10	-0.04	0.03	0.97	0.22

Table 4 shows the relationships among the 11 cognitive variables and three latent subgroups of PGD symptoms. Specifically, in the IBS with Class 1 as the reference group, a one-point increase in score increased the probability of a college student becoming Class 2 by 1.04 times and Class 3 by 1.09 times. With Class 2 as the reference group, a one-point increase in IBS score increased the probability of a college student becoming Class 3 by 1.05 times. For the BRIB and with Class 1 as the reference group, a one-point increase in score increased the probability of a college student becoming Class 2 by 1.58 times and Class 3 by 1.26 times. With Class 2 as the reference group, a one-point increase in BRIB score increased the probability of a college student becoming Class 3 by 1.25 times. However, there were differences in the relationships among the eight basic world assumptions of the WAS to the latent subgroups of PGD grief symptoms. Specifically, in terms of benevolence of the world, a one-point increase in score decreased the probability of a college student becoming Class 2 by 0.88 times and did not significantly change the probability of becoming Class 3, when using Class 1 as the reference group. A one-point increase in score decreased the probability of a college student becoming Class 3 by 1.12 times, when using Class 2 as the reference group. In terms of benevolence of people, using Class 1 as the reference group, a one-point increase in score decreased the probability of college students becoming Class 2 by 0.94 times (*p* = 0.07, borderline significant) and Class 3 by 0.76 times. Using Class 2 as the reference group, a one-point increase in score decreased the probability of college students becoming Class 3 by a factor of 0.81. For justice, with Class 1 as the reference group, a one-point increase in score increased the probability of college students becoming Class 2 by 1.12 times and Class 3 by 1.14 times. No other significant differences were found. For control, with Class 1 as the reference group, a one-point increase in score increased the probability of college students becoming Class 2 by 1.09 times and Class 3 by 1.15 times. With Class 2 as the reference group, a one-point increase in score increased the probability of college students becoming Class 3 by 1.05 times. For randomness, with Class 1 as the reference group, a one-point increase in score increased the probability of a college student becoming Class 2 by 1.24 times and Class 3 by 1.07 times. With Class 2 as the reference group, a one-point increase in the randomness score increased the probability of a college student becoming Class 3 by 0.86 times. For self-worth, with Class 1 as the reference group, a one-point increase in score decreased the probability of a college student becoming Class 2 by 0.82 times and Class 3 by 0.66 times. With Class 2 as the reference group, a one-point increase in self-worth decreased the probability of a college student becoming Class 3 by 0.80 times. For self-controllability, with Class 1 as the reference group, a one-point increase in score increased the probability of a college student becoming Class 2 by 1.08 times and Class 3 by 1.12 times. With Class 2 as the reference group, a one-point increase in score decreased the probability of a college student becoming Class 3 by 1.03 times (*p* = 0.09, borderline significant). If the controllability score increased by 1 point, college students were 1.03 times more likely to be Class 3. Gender and luck, as measured by the WAS, were not significantly associated with latent subgroups of PGD symptoms.

After synthesizing the results of the aforementioned multinomial logistic regression analysis and referring to previous research (Johnson and Lebreton, 2016), the relationships among the different cognitive variables and latent subgroups of PGD symptoms were generalized. First, the positive and negative coefficients were used to indicate the directions of the relationships between the cognitive variables and latent subgroups of PGD symptoms (positive numbers indicating positive relationships and negative numbers indicating negative relationships). The scores of the two irrational beliefs scales (i.e., IBS and BRIB) were significantly positive related to PGD symptoms; however, there were variations in the nature of the relationships between the different world assumptions and PGD symptoms, with justice, control, randomness, and self-controllability having a significantly positive relationship with PGD symptoms and benevolence of the world, benevolence of people, and self-worth having a significantly negative one. Second, the magnitudes of the effect sizes of the relationships among the cognitive variables and latent subgroups of PGD symptoms were indicated by the magnitudes of the OR coefficient (with larger values indicating closer relationship effects between variables). It was found that the OR coefficient of the BRIB was the largest among all the variables, indicating that its relationship with different subgroups of PGD symptoms was the greatest.

## Discussion

Based on an individual-centered perspective, this study explored the latent subgroups of PGD symptoms experienced by Chinese college students and examined the relationships among cognitive variables (i.e., irrational beliefs and basic world assumptions) and various subgroups of PGD symptoms. The goal was to promote a better understanding of the relationship between PGD symptoms and cognitive variables among Chinese college students.

### Latent subgroups and the characteristics of PGD symptoms in Chinese college students

This study found that PGD symptoms among Chinese college students can be divided into three latent subgroups: mild, moderate, and severe. Specifically, the mild PGD symptoms group accounted for 31% of the total, and the total TGI score for this group was the lowest. However, it was still higher than 1 (rarely) for the “longing” symptom, indicating that bereavement does not completely correlate with PGD symptoms, or at least “longing.” It may still be associated with other symptoms of PGD. The moderate PGD symptoms group accounted for 48% of the total. The total TGI score for this group was in the middle of the three groups, and most of the PGD symptoms ranged between 1 (rarely) and 2 (sometimes), indicating that most of the college students had some PGD symptoms after bereavement, especially the four symptoms of “longing,” “anger,” “reminders,” and “bitterness.” The severe PGD symptoms group accounted for 21% of the total. This group had the highest total TGI score, with most of the PGD symptoms higher than 2 (sometimes). Some were even higher than 3 (often), indicating that this group was more maladjusted after bereavement, especially in the following areas: “longing,” “anger,” “reminders,” and “bitterness.” This was especially true for “longing,” “reminders,” and “bitterness.” Although the three subgroups of PGD symptoms among Chinese college students shared some similar characteristics in terms of expression (for example, “longing,” “reminders,” and “bitterness” were PGD symptoms that all three subgroups exhibited); these three subgroups had similar patterns of symptoms. In other words, there was no intersection between the PGD symptoms of these three potential subgroups, and the morphologic trends were relatively consistent. The subgroups mainly significantly differed in terms of total TGI scores. The similarity in PGD symptoms among Chinese college students may be related to the limited distribution of loss events experienced by this group. As described in the introduction, the study group had been living in a school area for a long period of time, and the important influential factors of PGD symptoms (such as the object and cause of the loss experienced) were relatively consistent (Ehlers, 2006; Kristensen et al., 2012), likely influencing the PGD symptoms to be similar. This result corresponds with the assumption that PGD is a syndrome created by a number of co-occurring symptoms of persistent grief (Prigerson et al., 2009). The differences in severity of PGD symptoms may be related to differences in students’ cognitive belief systems. To summarize the previous analyses, this study concluded that PGD symptoms among Chinese college students can be divided into three subgroups, and the differences among these subgroups mainly manifest in the overall severity of PGD symptoms.

### Cognitive variables as predictors of latent categories of PGD symptoms in Chinese college students

This study found that two cognitive variables related to bereavement (irrational beliefs and basic world assumptions) were significantly correlated to Chinese college students’ PGD symptoms, indicating an association between the severity of college students’ PGD symptoms and cognitive variables. This result supports the Cognitive-Behavioral Conceptualization Model’s expectation from an empirical perspective (Boelen et al., 2006). Because the participants of this study are Chinese college students (of an Asian cultural background), the irrational beliefs related to individual bereavement should be a key factor in the degree of PGD symptoms, thus providing further evidence of the universality of the CBCM’s basic views.

This study found that the two cognitive variables of irrational beliefs and basic world assumptions had a significant correlation with Chinese college students’ PGD symptoms, of which the coefficient of correlation (OR value) between irrational beliefs and PGD symptom severity was the largest. This suggests that bereavement-related irrational thinking may be a primary factor associated with the severity of PGD symptoms. For instance, bereaved individuals may believe that they are unable to cope with life without the deceased (i.e., low frustration tolerance). To some extent, this result supports Ellis’s (2001) view that people can choose to respond to traumatic events (including bereavement) with appropriate or unreasonable emotional and behavioral responses. In other words, irrational thinking is a significant factor in dealing with the impact of traumatic life events (Ellis, 2001). It follows that after experiencing a bereavement event, those individuals who hold irrational beliefs regarding the bereavement event will exhibit additional irrational beliefs or behaviors such as catastrophizing and low frustration, and devaluing beliefs such as “I feel worthless since he/she has passed away.” This type of outcome may be related to the exacerbation of bereavement symptoms and could even produce psychopathological reactions such as PGD symptoms (Malkinson and Ellis, 2000; David et al., 2002; DiLorenzo et al., 2007). At the same time, previous studies have also found that there is a significant relationship between individual irrational beliefs and other negative symptoms after bereavement, such as depressive- and anxiety-related indicators (Malkinson and Ellis, 2000; Boelen et al., 2003; Nagy and Szamosközi, 2014).

Secondly, there was also a statistically significant relationship between basic world assumptions and the severity of PGD symptoms. According to Janoff-Bulman (1989), individuals form a set of basic world assumptions about the self, others, and the world within a specific cultural environment. It is a cognitive schema necessary for people’s daily functioning and provides an unassailable illusion. Bereavement events can lead to the deterioration of the basic world assumptions previously held by individual. For example, previous studies based on variables have found that compared with individuals who have not experienced bereavement, those with bereavement experience showed significant differences in meaningfulness of the world (e.g., justice, control, randomness) and worthiness of the self (e.g., self-worth, self-controllability). If other aspects of their world assumptions have been damaged (Faschingbauer et al., 1977; Schwartzberg and Janoff-Bulman, 1991; Boelen et al., 2004; Zukerman and Korn, 2014; Huang et al., 2023), the breakdown of these basic world assumptions will correlate with the individual suffering from PGD symptoms. It was not found that benevolence of the world (e.g., benevolence of people) was damaged by bereavement events. The results of the present research show that the nature of the correlation effect of the eight basic world assumptions on the severity of PGD symptoms was different for different dimensions. This study had findings similar to those of previous studies (Schwartzberg and Janoff-Bulman, 1991; Boelen et al., 2004; Zukerman and Korn, 2014; Huang et al., 2023). Specifically, justice, control, randomness, and self-controllability are positively associated with the severity of PGD symptoms. Thus, the greater the degree to which an individual suffers from these four convenient basic world assumptions, the more severe their PGD symptoms will be. This result, together with those of previous studies, supports the notion that bereavement is associated with the breakdown of two basic world assumptions: the meaningfulness of the world (e.g., justice, control) and worthiness of the self (e.g., self-control). For example, bereaved individuals may perceive life as full of uncertainty and determined by chance and may resort to more irrational self-control. This can cause the individual’s mental representations of the external world, self, and death to appear incongruent or even contradictory to their original assumptions about the world, potentially worsening their PGD symptoms (Boelen et al., 2006). The results further suggest that an individual’s increased vulnerability to these basic world assumptions can exacerbate the severity of PGD symptoms.

Interestingly, this study also found that the three assumptions of benevolence of the world, benevolence of people, and self-worth significantly negatively associated with the severity of PGD symptoms in Chinese college students. In other words, the higher the scores for these three basic world assumptions, the lower the severity of the PGD symptoms, from which we hypothesized that these three basic world assumptions were related to a reduction in the severity of PGD symptoms and could serve as variables protecting against such symptoms. This result has not been found in previous studies on bereaved people in Western countries; such studies did not find that there would be changes in the two dimensions of benevolence of the world and people in terms of individuals’ basic world assumptions after bereavement (Boelen et al., 2004). These two basic world assumptions have not been found to have a significant correlation with PGD symptoms (Schwartzberg and Janoff-Bulman, 1991). Therefore, this study speculates that the relationship between individual basic world assumptions and bereavement events may vary depending on culture, particularly in Eastern cultures. Why did this study find that the three basic world assumptions of benevolence of the world, benevolence of people, and self-worth had significant negative associations with the symptoms of PGD among Chinese college students? This result may be related to funeral etiquette in the Chinese culture. Chinese people hold a unique funeral after someone dies. For example, relatives and friends, fellow villagers, and neighbors will almost spontaneously carry out collective mourning for the bereaved family, send consolation money, and so on, in order to express their condolences and concern, practices unique to Chinese funeral culture. This is also a way to provide social support, making the bereaved feel the respect and care of important individuals and groups and showing them that the “world is full of care” and “human beings are friendly and moral.” Thus, they experience a sense of collective belonging that strengthens the relationship within the social support system such that the bereaved individual can experience it. In such cases it is easier to evaluate oneself positively and experience the power of oneself, and the direction and goals of life become more clear, helping people maintain their sense of the benevolence of the world and enhancing their self-worth (Zheng et al., 2016; Li et al., 2018).

### Application value, limitations, and future research directions

This study explored the relationship between post-bereavement cognitive variables and PGD symptoms in Chinese college students. The results showed that both irrational beliefs and basic world assumptions of bereaved individuals had a strong connection to the severity of PGD symptoms. These results will be of great value to those pursuing psychological interventions for this cohort.

This study argues that psychological interventions for PGD symptoms in Chinese college students should use cognitive-behavioral therapy (CBT) because the results support the existence of significant associations between cognitive variables and PGD symptoms; this is consistent with the core logic of CBT (Ellis, 2001; Rubin et al., 2016). Since among these cognitive variables, bereavement-related irrational beliefs had the highest correlation with PGD symptoms (the largest OR coefficient) (Duffy and Wild, 2017; Boelen et al., 2021), this suggests that the irrational beliefs of Chinese PGD may be the primary variable of concern for counselors. According to the CBT model, when intervening on behalf of Chinese college students with PGD symptoms, counselors first need to think adaptively about the irrational beliefs contributing to their symptoms and adjust their tendency to engage in automated irrational thinking. Secondly, it is also necessary to adjust any basic world assumptions related to PGD symptoms, as those assumptions are the core beliefs motivating PGD symptoms. Those suffering from such symptoms should be helped to reorganize their belief systems about themselves and the world. It should be added that in the behavioral therapy stage of CBT, counselors should help them build their internal and interpersonal resources, focusing on the protective correlation effect of the three assumptions of benevolence of the world, benevolence of people, and self-worth. For example, we suggest that that the bereaved actively participate in funeral rituals such as the annual Qingming Festival. Ultimately, this will help them change their negative thoughts and beliefs, reconstruct the belief system about themselves and the world, eliminate emotional problems, and achieve a higher rate of success for interventions related to PGD.

This study does have some limitations that require the attention of future research. First, the TGI was used to measure the symptoms of PGD in Chinese college students. Although the measure can reflect such symptoms in college students, it is still somewhat outdated, and future work should use newer tools to measure the symptoms of this disorder, such as the Prolonged Grief 13-Revised (Prigerson et al., 2021). Secondly, the sample source for the study was made up entirely of college students (whose bereavement experience is limited), and different causes of death will have correlations with distinct cognitive modifications (Silverman and Rubin, 2015). Future research should expand the distribution of samples (Djelantik et al., 2017). Third, for the results of the multicollinearity analysis of the 11 predictor variables, it is important to note that the VIF index value for benevolence of the world (BoW) was 3.147, which is greater than 2.5. BoW is uniquely significant in the Chinese culture because benevolence has for thousands of years been one of the core ideas expressed in Chinese Confucianism, and Chinese benevolence is embodied both by individual Chinese people and the collective Chinese culture. Thus, the important cognitive variable of BoW was not excluded. However, this may have had an impact on the results of the multiple regression analysis, which is a concern. Fourth, this study did not take into account non-cognitive variables that are known to be associated with PGD, so it is impossible to determine if cognitive variables are more important than those that are attachment-related or interpersonal. To gain a better understanding of Chinese college students’ PGD symptoms and their association with cognitive variables, future research should consider both cognitive and attachment-related factors, such as attachment styles (Sekowski and Prigerson, 2022b), the quality of the relationship prior to loss (Sekowski and Prigerson, 2022a), excessive interpersonal dependency (Sekowski and Prigerson, 2021), and maintaining a connection with the deceased (Sekowski, 2021). Lastly, a cross-sectional research method was used in this study, which has some shortcomings. The relationship between the cognitive variables and symptoms of PGD is not only a state relationship, but also a dynamic process of inner reorganization and balance. Thus, future research should use longitudinal and qualitative research methods. This will better explain the relationship between the cognitive variables and PGD symptoms (Zhou and Jia, 2021), such as the positive correlation of the two world assumptions of benevolence of the world and benevolence of people with PGD symptoms.

## Conclusion

Based on the above discussion, this study concluded that the PGD symptoms of Chinese college students can be classified into three subgroups: mild, moderate, and severe. All cognitive variables (e.g., irrational beliefs, basic world assumptions, etc.) have significant relationships with PGD symptoms in Chinese college students, among which bereavement-related irrational beliefs were found to have the largest positive correlation. This study was also the first to find that the nature of the relationship between basic world assumptions and Chinese college students’ PGD symptoms varied across dimensions. Specifically, justice, control, randomness, and self-controllability were significantly positively related to PGD symptoms, whereas benevolence of the world, benevolence of people, and self-worth were significantly negatively associated with such symptoms and may act as variables protecting against them. These results have practical value for CBT-based interventions for PGD-related problems in Chinese college students.

## Data availability statement

The original contributions presented in this study are included in this article/supplementary material, further inquiries can be directed to the corresponding authors.

## Ethics statement

The studies involving humans were approved by the Biomedical Research Ethics Committee of Fujian Medical University (Nos. 2023-121). The studies were conducted in accordance with the local legislation and institutional requirements. The participants provided their written informed consent to participate in this study.

## Author contributions

The idea for this study was conceived and revised the manuscript by FH, ML, and WT. WT and YC collected the data. FH and WT engaged in the analysis and interpretation of the data and wrote the manuscript. All authors contributed to the article, designed the research, reviewed, read, and approved the submitted version of the manuscript.
